# Supplementary material for: Phylogenetic Diversity of Lhr Proteins and Biochemical Activities of the Thermococcales aLhr2 DNA/RNA Helicase
Source: Biomolecules. 2021 Jun 26;11(7):950. doi: 10.3390/biom11070950 (PMC8301817; doi:10.3390/biom11070950)
Supplement: Supplementary file 1 [file biomolecules-11-00950-s001.zip › HAjj etal biomolecules suppl files -V1806.pdf]

Phylogenomic diversity of Lhr-proteins and biochemical activities of the Thermococcales aLhr2 DNA/RNA helicase

Hajj et al

Supplementary Material (Figures & Tables)

Supplementary Figures

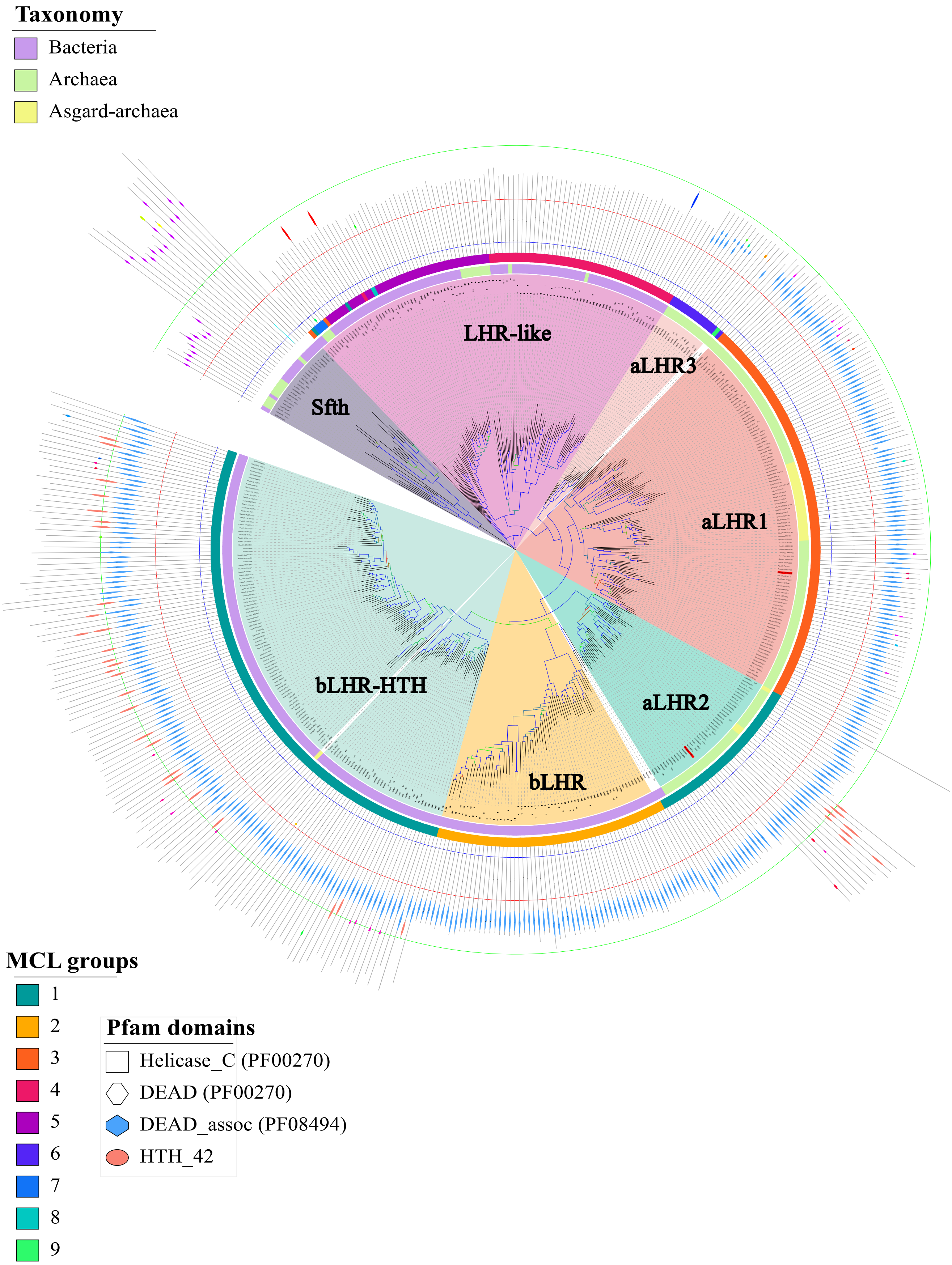

Figure S1: Phylogenetic tree of Lhr sequences rooted with Sfth representative proteins. The Sfth helicases being the closest related family of Lhr helicases, 24 representative sequences have been used as outgroup to root the tree of the Lhr family sequences. Legend as in Figure 2.

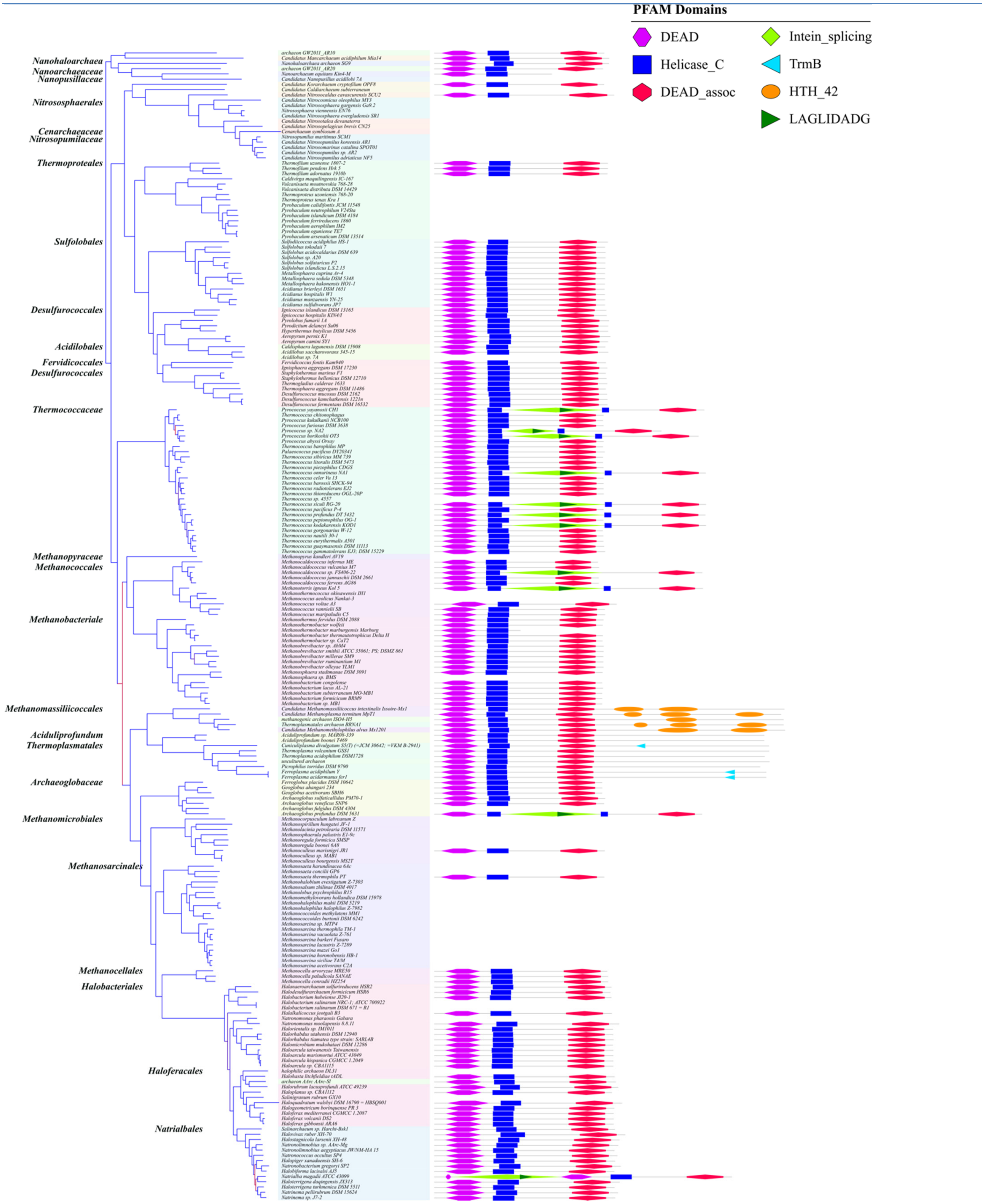

Figure S2: Domain architecture of aLhr2 proteins. Left panel: species tree of the Archaeal genomes as in Figure 3. NCBI taxonomy was reported at the order or family level. The right panel shows the motif architecture of the protein. Each protein is represented by a black line on which the Pfam domains have been mapped. The LAGLIDADG domain is integrated in the Intein_splicing domain (see the legend for the domain colours). The figure was built with iTOL.

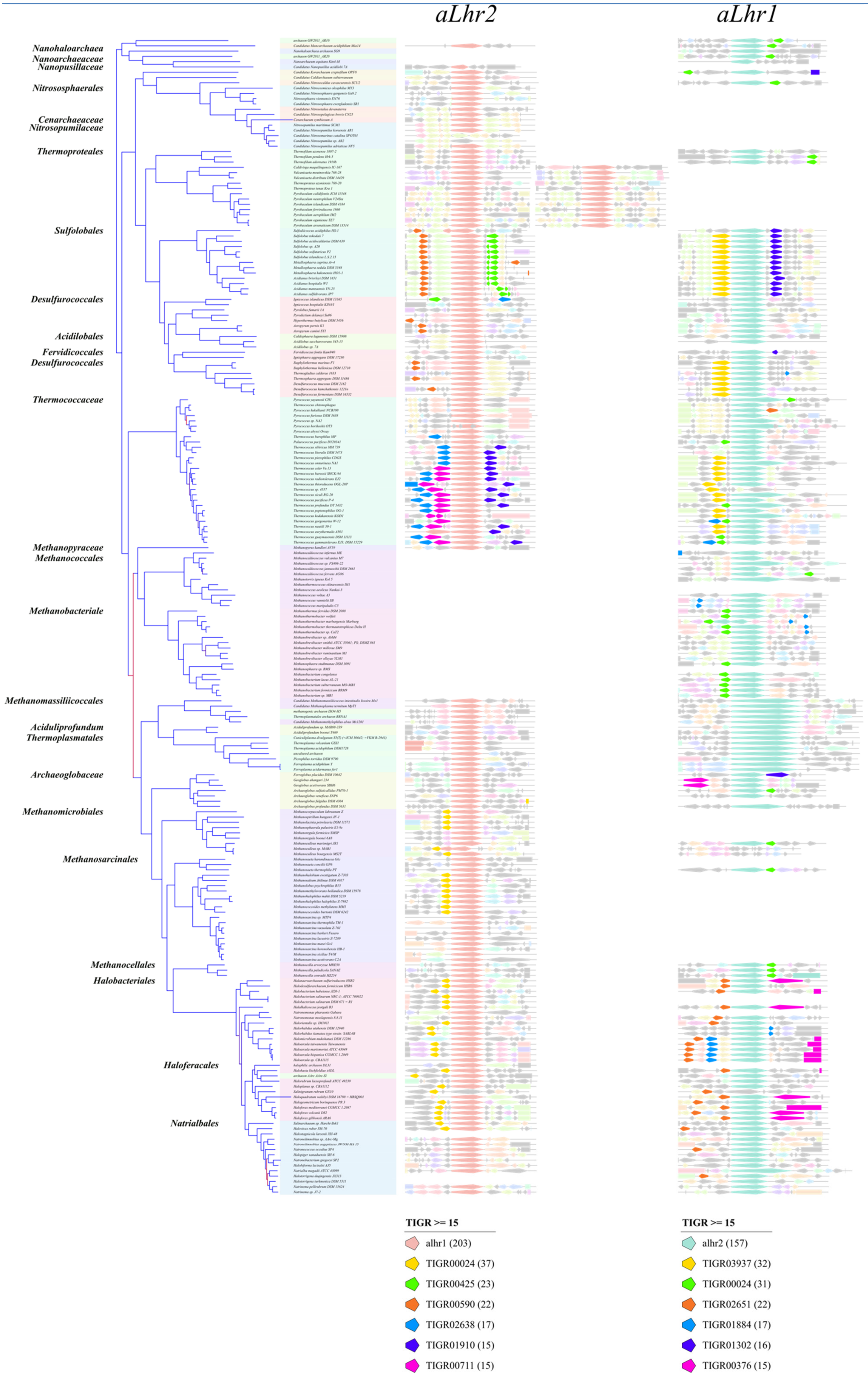
aLhr2
aLhr1
Nanohaloarchaea
Nanoarchaeaceae
Nanopusillaceae
Nitrososphaerales
Cenarchaeaceae
Nitrosopumilaceae
Thermoproteales
Sulfolobales
Desulfurococcales
Acidilobales
Fervidicoccales
Desulfurococcales
Thermococcaceae
Methanopyraceae
Methanococcales
Methanobacteriale
Methanomassiliicoccales
Aciduliprofundum
Thermoplasmatales
Archaeoglobaceae
Methanomicrobiales
Methanosarcinales
Methanocellales
Halobacteriales
Haloferacales
Natrialbales
TIGR >= 15
alhr1 (203)
TIGR00024 (37)
TIGR00425 (23)
TIGR00590 (22)
TIGR02638 (17)
TIGR01910 (15)
TIGR00711 (15)
TIGR >= 15
alhr2 (157)
TIGR03937 (32)
TIGR00024 (31)
TIGR02651 (22)
TIGR01884 (17)
TIGR01302 (16)
TIGR00376 (15)

Figure S3: The neighbourhood of aLhr1 and aLhr2 encoding genes. Left panel: species tree of the Archaeal genomes as in Figure 3. For the two panels, the annotation of the genes located at 4000 bp upstream and downstream of the reference gene (*alrh1* or *alhr2*) was extracted. Genes with one end outside these boundaries are represented by rectangles. The genes are stained according to the TIGR annotation. TIGR annotations with occurrences greater than or equal to 15 are flagged with a flashy color (color code at the bottom of each gene context) and low frequency occurrences are colored with very light pastel colors to highlight conservation limited to closely related species. *alhr1* genes have not been annotated in *Candidatus Nitrosopumilus sp. AR2, Candidatus Nitrosomarinus catalina SPOT01 and Salinigranum rubrum GX10* and *alhr2* gene has not been annotated in *Salinigranum rubrum* GX10. Gene fissions are present in *Methanoculleus sp.* MAB1 (*aLhr1*) and in *Methanothermobacter thermautotrophicus* Delta H (*aLhr2*). Three gene contexts have been conserved but without the *alhr2* gene in *Thermococcus sp*. 4557, *Methanoculleus sp.* MAB1 and *Methanoculleus bourgensis* MS2T. The figure was built with iTOL.

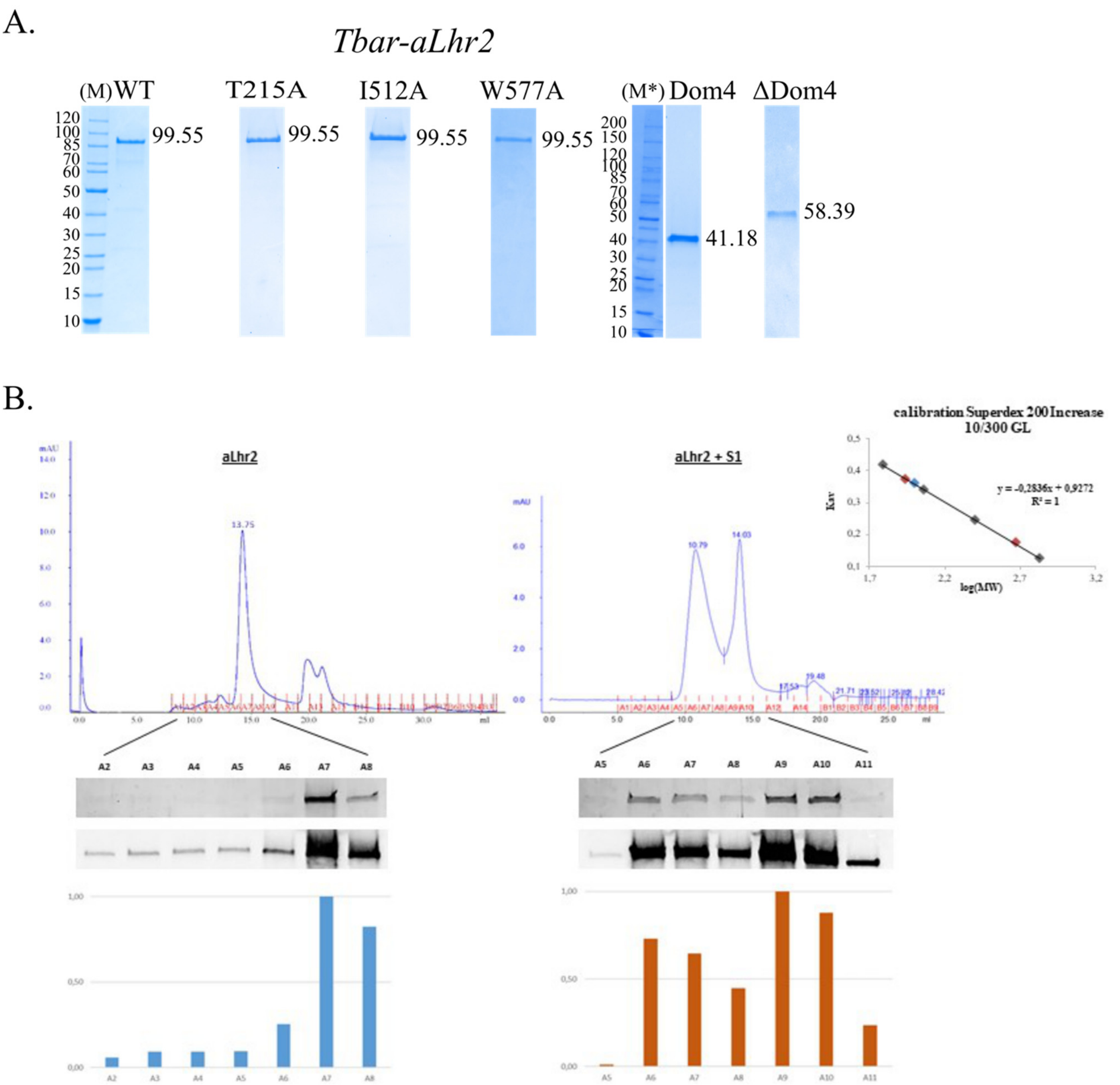

Figure S4: Purification and oligomeric state of *Tbar*-aLhr2. (**A**) Recombinant proteins used in this study. Highly purified *Tbar*-aLhr2 WT and variants obtained by two-step purification were separated by SDS-PAGE and visualized by Coomassie blue staining. The predicted molecular weight of each protein is indicated. (**B**) Left panel: Size exclusion chromatography (SEC) shows that in solution *Tbar*-aLhr2 WT is a monomeric protein with a single peak at an elution volume (Ve) of 13.75mL that corresponds to an apparent molecular mass of 100.5kDa. Right panel: SEC shows that, in presence of DNA_{50} (S1), *Tbar*-aLhr2 WT assembles as a multimer (Ve of 10.79mL). Based on column calibration, the multimer seems to be constituted of 4 to 5 molecules of *Tbar*-aLhr2. For both panel, the SEC elution curves, the Coomassie blue SDS-PAGE and the Western blotting of *Tbar*-aLhr2 are given. The relative quantifications of the anti-aLhr2 Western blot signals are plotted. The S200 increase 10/300 GL column was calibrated with Ferritin (440kDa), Aldolase (158kDa), Conalbumin (75Kda) and Ovalbumin (43kDa). The calibration curve is shown as the top of the right panel.

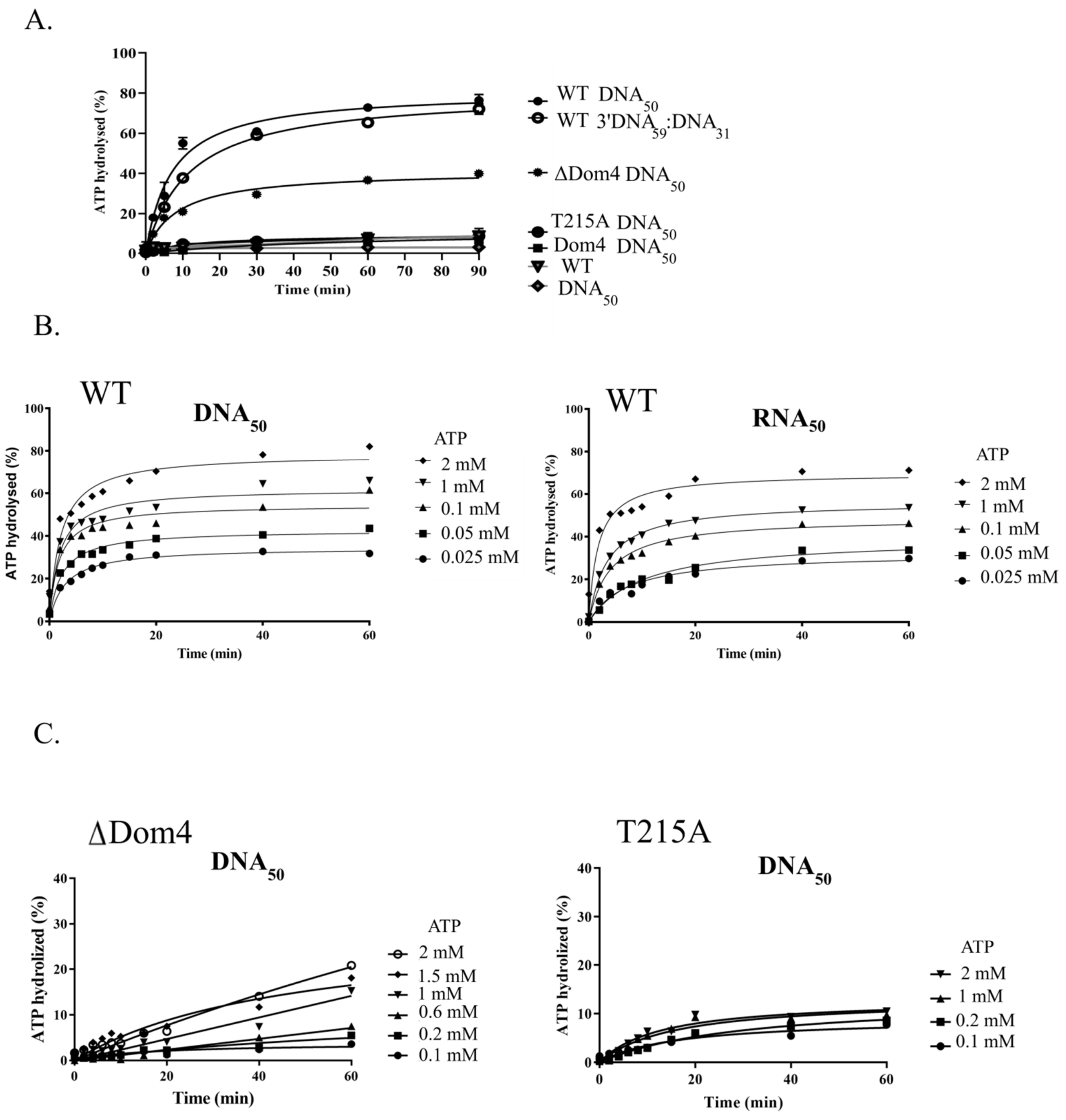

Figure S5: ATPase activity of WT and derivative*Tbar*-aLhr2. (A) ATPase activities of *Tbar*-aLhr2-WT, *Tbar*-aLhr2-ΔDom4 and *Tbar*-aLhr2-Dom4 in the presence of DNA_{50} or 3'DNA_{59}:DNA_{31}. Same controls as in Figure 5 with no protein (DNA_{50}). (B) Kinetics of ATP hydrolysis as in (A) using a range of ATP concentration from 0.1mM to 2mM as indicated, in presence of single stranded nucleic acid DNA_{50} (left panel) or RNA_{50} (right panel) (C) ATPase activities of *Tbar*-aLhr2-ΔDom4 and *Tbar*-aLhr2-T215A derivatives using a range of ATP as in (B), in presence of single stranded nucleic acid DNA_{50}.

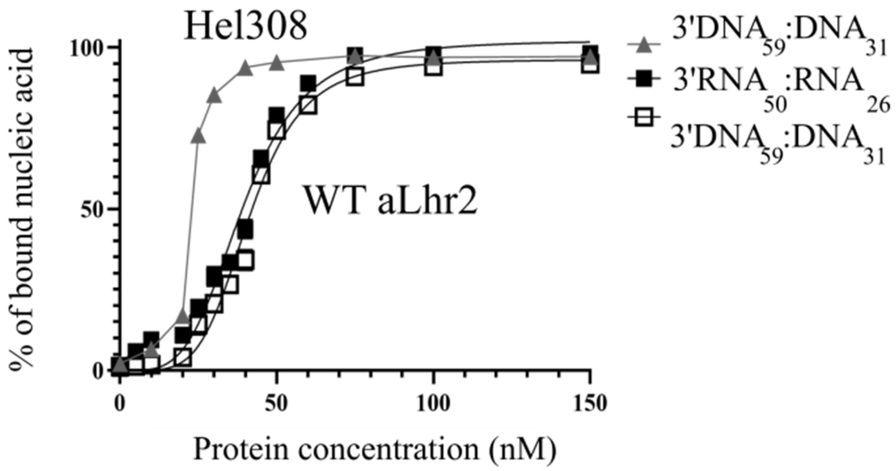

Figure S6: Binding affinity of *Tbar*-aLhr2 and *Paby*-Hel308 for 3'overhang DNA and RNA duplexes as indicated. Legend as in Figure 6.

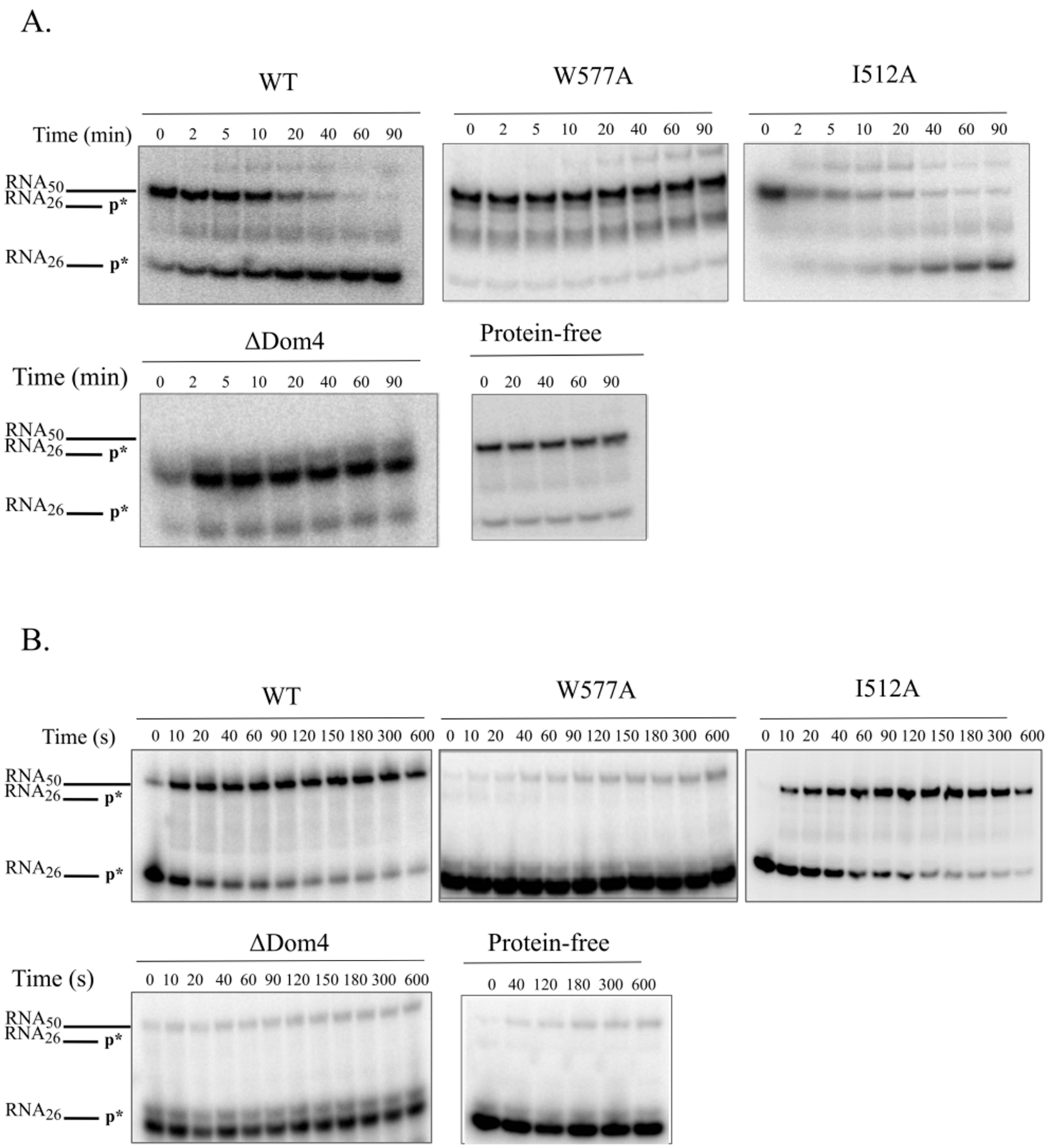

Figure S7: Wild type *Tbar*-aLhr2 and variants unwinding and annealing activities. (**A**) Unwinding activities: a pre-formed 3'DNA_{50}:RNA_{26} radiolabelled hetero-duplex was incubated for 0, 2, 5, 10, 20, 40, 60 and 90 min at 65°C with 5mM of ATP and 1µM oligoTrap in presence or absence of 250nM of *Tbar*-aLhr2WT. Reaction products without (protein-free) or with protein were separated on a native 8% acrylamide gel; **(B)** Annealing activities: the radiolabelled RNA_{26} was incubated with the unlabelled 3'DNA_{50} from 10, 20, 40, 60, 90, 120, 150, 180, 300 and 600 seconds at 65°C with or without 250nM of *Tbar*-aLhr2. Product reactions without protein (free-protein) or with protein were separated on a native 8% acrylamide gel;

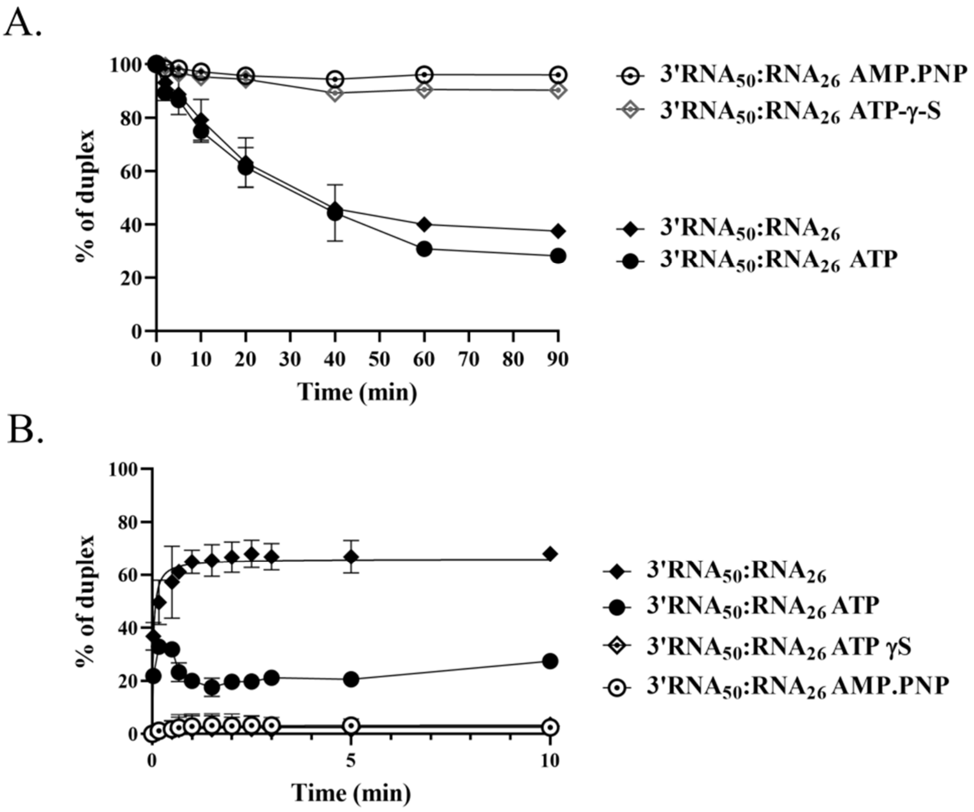

Figure S8: Unwinding and strand-annealing activities of *Tbar*-aLhr2WT in presence of ATP, AMP.PNP and ATPγS (ATP analogues). (**A**) Kinetics of strand dissociation of 3'RNA$_{50}$:RNA$_{26}$ duplex; (**B**) Kinetics of strand annealing formation of 3'RNA$_{50}$:RNA$_{26}$ duplex. Legend as in Figure 6 and 7, respectively.

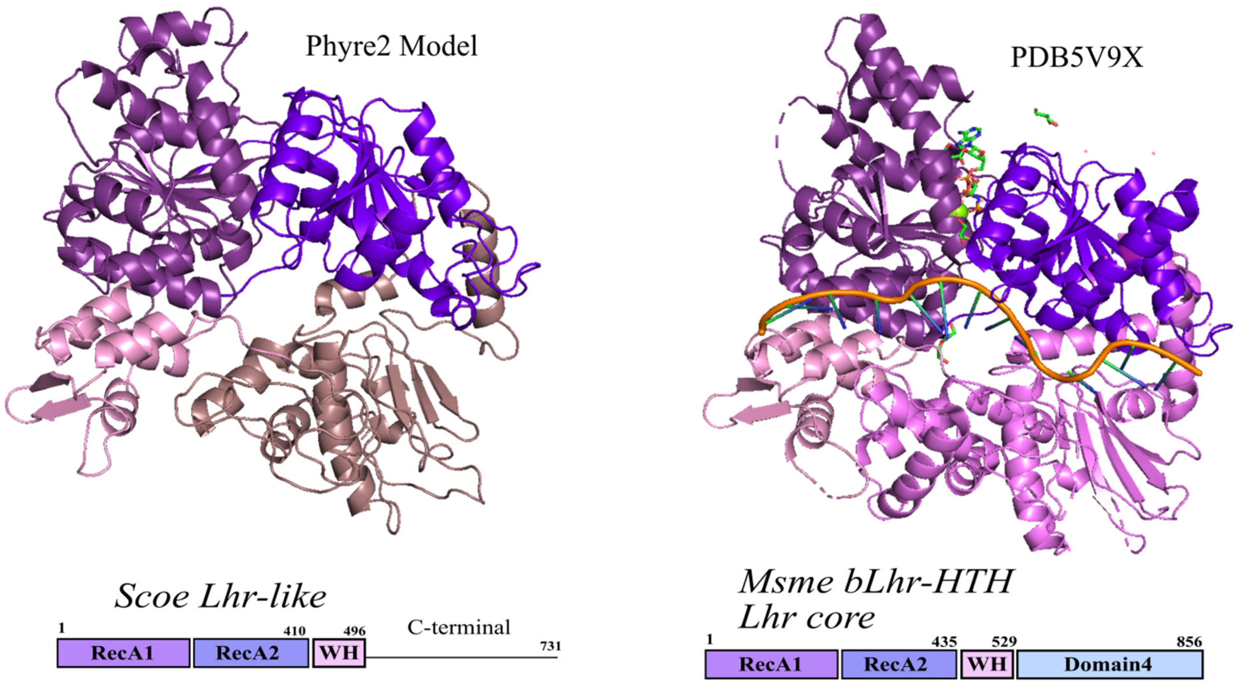

Figure S9: Structure models of *M. smegmatis* bLhr-HTH and *S. coelicolor* Lhr-like. The structure of the *M. smegmatis* Lhr-core (1-856) of bLhr-HTH (accession number - PDB5V9X) is shown on the right panel. A model of *S. coelicolor* Lhr-like (SCO6640), built using the Phyre2 server (http://www.sbg.bio.ic.ac.uk/phyre2), is shown on the left. The RecA1 domain is in violet-blue, RecA2 in purple-blue, WH domain in light pink, Domain 4 of *M. smegmatis* bLhr-HTH in pink and C-terminal domain of *S. coelicolor* Lhr-like in brown. The stereo view of the models was analysed using PyMOL software.

Supplementary Tables

Table S1: (His$_6$)-*Pab*y-ASH-Ski2 list of protein partners (from the most to the less specific) extracted from [18].

ID	ORF	Name	Protein Description	Ref. Spectra	Spec. Index
Q9V133	PAB2412		Uncharacterized protein	15.5	0
Q9UZ86	PAB2423	rgy	Reverse gyrase	14	0
Q9UY85	PAB1284	DHH-like nuclease	DHH family	9	0
Q9UY07	PAB1115	Nucleotidyl-transferase	Glucose-1-P thymidylyltransferase	8	0
G8ZI82	PAB1751	5'-3'exo-ribonuclease	aRNase J	8	0
G8ZJS6	PAB0744	Lhr2	Large helicase-related protein type2	6.5	0
Q9V114	PAB0424	Rpo1	DNA-directed RNA polymerase subunit 1'	6.5	0
Q9V113	PAB0425	Rpo1''	DNA-directed RNA polymerase subunit 1"	4	0
Q9UYS8	PAB1430	Topo1	DNA topoisomerase 1	3.5	0
Q9V1T8	PAB2122	L2	50S ribosomal protein L2	3.5	0
Q9V191	PAB0368	rps2	30S ribosomal protein S2	3.5	0
P61992	PAB0361	S4	30S ribosomal protein S4	3.	0
Q9V0E2	PAB0569	Malate dehydratase	3-isopropylmalate dehydratase	2.5	0
Q9UXX7	PAB1136	Rpp30	Ribonuclease P protein component 3	2.5	0
Q9V181	PAB1136	Rpp30	Ribonuclease P protein component 3	2.5	0
Q9UYS7	PAB1429		Uncharacterized protein	2	0
G8ZHR3	PAB0190	Hef nuclease	ERCC4-like helicase	2	0
Q9V116	PAB7151	Rpo5	RNA polymerase subunit 5/ dsDNA binding	2	0
Q9V1U8	PAB2397	S4e	30S ribosomal protein S4e	1.5	0
Q9UZL4	PAB0749	S8e	30S ribosomal protein S8e	1.5	0
Q9UZP0	PAB0732	Rpo4	DNA-directed RNA polymerase, subunit 4	1.5	0
Q9UZX2	PAB1688		Uncharacterized protein	1.5	0
Q9V0H2	PAB1817	Lhr1	Large helicase-related protein type 1	1.5	0
G8ZHS0	PAB2163	RPA41	Replication factor A (RPA41)	22	0.01
Q9V1V6	PAB2137	L30	50S ribosomal protein L30P	4	0.02
Q9V0G8	PAB1813	S19e	30S ribosomal protein S19e	2.5	0.05
Q9V2M1	PAB2313		ATP-dependent RNA helicase	291.5	0.05
Q9V1Z1	PAB2165	RPA32	Replication factor A (RPA32)	16.	0.07
Q9UXS5	PAB1167	L10	50S ribosomal protein L10	1.5	0.09
Q9V196	PAB0365	L13	50S ribosomal protein L13P	3.5	0.13
Q9V115	PAB0423	Rpo2	DNA-directed RNA polymerase subunit 2	25	0.14
Q9UZN6	PAB1633	PINA	ATPase	4	0.14
Q9UZD0	PAB0810		Predicted ATPase	6	0.23
Q9V1V5	PAB2136	S5	30S ribosomal protein S5	4.5	0.24
Q9V089	PAB2390	Sun domain	Sun protein (Fmu) S-adenosyl methionine/RNA binding domain	5	0.27
Q9V2L4	PAB2305	Nop5	Component of RNA methylating RNP complex (C/D box)	13.5	0.29
P62008	PAB0460	L7Ae	50S ribosomal / C/D RNP protein L7Ae	3.5	0.34
Q9V1U6	PAB2436	L14	50S ribosomal protein L14	2	0.35
Q9V1F2	PAB0316	DNaG	Exosome component cap subunit	11.5	0.43
Q9V192	PAB0367	Enolase	Eno-like enolase related/glycolyse pathway	21	0.45
Q9UZ78	PAB2428	TmcA	tRNA(Met) cytidine acetyltransferase TmcA	35	0.58
Q9V1U7	PAB2128	L24	50S ribosomal protein L24	2.5	0.71
Q9V2L5	PAB2306	Fibrillarin	rRNA/tRNA 2'-O-methyltransferase	11	0.73
Q9V1T5	PAB2120	L3	50S ribosomal protein L3	4.5	0.85

Paby-aLhr2 protein is highlighted in grey. The partners were identified by pulldown-MS/MS as described in [18]. Recombinant ASH-Ski2 protein tagged as its N-terminus was used as bait protein in *P. abyssi* cellular extract. The MS proteomics data are available at the ProteomeXchange Consortium via the PRIDE partner repository "PXD015856". Shortly, the MS data from three replicates were processed in order to identify specific interaction signals. MS data from control samples with no-bait proteins were also generated. Global specific spectra from samples were normalized between replicate series and a cut-off of two normalized spectra as minimum MS signal for network hit validation was used. Normalized spectra were then averaged between replicates and referenced versus control to calculate the number of 'Referenced Spectra'. Calculation of the 'Specificity Index' score is the ratio of the averaged normalized spectra in control versus assay. The 'Specificity Index' varies from 0 to 1 (with a maximum threshold of 1) as the specificity decreases.

Table S2: The Lhr and Sfth protein sequence identifiers used in Figures 2 and Supplementary Figure S1, with the related organisms, Uniprot accession numbers and locus-tags of the achaeal Lhr (Excel file Table S2A), bacterial Lhr (Excel file Table S2B) and Sfth (Excel file Table S2C).

Table S3: Sequences of synthetic oligonucleotides used in this study.

Primers	Sequences (5'-3')	Purpose
B15-1	CATATGTATATCTCCTTCTTAAAGTT	Antisense oligo to linearize pET11b and to construct pET11b-aLhr2-Dom4 by inverse PCR from pET11-aLhr2-WT
B13-18	GGATCCGGCTGCTAACAAAGCC	Sense oligo to linearize pET11b
B18-2	TTAGCAGCCGGATCCTCATTCAAGCTCCCCGATCA	Sense oligo to amplify *Tbar*-aLhr2 from genomic DNA and clone it into pET11b
B18-4	TTAGCAGCCGGATCCTCATTCAAGCTCCCCGATCA	Antisense oligo to amplify *Tbar*-aLhr2 from genomic DNA and clone it into pET11b
B18-9	TGGAATTGTACCCGTGTTCATG	Sense oligo to construct pET11-aLhr2-ΔDom4 by inverse PCR from pET11b-aLhr2-WT
B17-33	[Phosphate]-TAACAAAGCCCGAAAGGAAGCT	Antisense oligo to construct pET11b-aLhr2-ΔDom4 by inverse PCR from pET11-aLhr2-WT
B18-10	[Phosphate]-GATGAGGCTAAAATCGAAGTTTA	Sense oligo to construct pET11b-aLhr2-Dom4 by inverse PCR from pET11-aLhr2-WT
B19-04	GATCCCAGCGGCGTTTTCTGAAATGTTGCCT	Sense oligo to introduce the W577A mutation in pET11b-aLhr2 by directed mutagenesis
B19-05	TTCAGAAAACGCCGCTGGGATCGTTGGCTT	Antisense oligo to introduce the W577A mutation in pET11b-aLhr2 by directed mutagenesis
B19-06	AACACGGGTACAGCTCCAGATGAGGCTAAA	Sense oligo to introduce the I512A mutation in pET11b-aLhr2 by directed mutagenesis
B19-07	ATCTGGAGCTGTACCCGTGTTCATGTAGTA	Antisense oligo to introduce the I512A mutation in pET11b-aLhr2 by directed mutagenesis
B19-22	AACCTCTTCGAGGGGATGAATAGCGGCACT	Sense oligo to introduce the T215A mutation in pET11b-aLhr2 by directed mutagenesis
B19-20	TTCGTTAGAATCGGTCTCAGTGCCGCTATTC	Antisense oligo to introduce the T215A mutation in pET11b-aLhr2 by directed mutagenesis
B15-37	CCGGAATTCTAATACGACTCACTATAGATCGATAGTCT CTAGACAGCATG	Sense oligo to assemble T7-RNA$_{50}$ PCR product that is used for *in vitro* transcription of RNA$_{50}$
B15-38	ACGCTGCCGAATTCTGGCTTGCTAGGACATGCTGTCT AGAGACTATCG	Antisense oligo to assemble T7-RNA$_{50}$ PCR product that is used for *in vitro* transcription of RNA$_{50}$

Table S4 : Nucleotide sequences of the single-stranded and duplex DNA and RNA substrates used in this study.

DNA & RNA substrates	
DNA$_{50}$	5' ATCGATAGTCTCTAGACAGCATGTCCTAGCAAGCCAGAATTCGGCAGCGT
RNA$_{50}$	5' AUCGAUAGUCUCUAGACAGCAUGUCCUAGCAAGCCAGAAUUCGGCAGCGU
DNA$_{59}$	5' GACGCTGCCGAATTCTACCAGTGCCTTGCTAGGACATCTTTGCCCACCTGCAGGTTCAC
DNA$_{26}$	5' TAGCTATCAGAGATCTGTCGTACAGG
RNA$_{26}$	5' ACGCUGCCGAAUUCUGGCUUGCUAGG
DNA$_{31}$	5' CTGCGACGGCTTAAGATGGTCACGGAACGAT
5'DNA$_{59}$:DNA$_{31}$	5' GACGCTGCCGAATTCTACCAGTGCCTTGCTAGGACATCTTTGCCCACCTGCAGGTTCAC<br>CTGCGACGGCTTAAGATGGTCACGGAACGAT 5'
3'DNA$_{50}$:RNA$_{26}$	5' ATCGATAGTCTCTAGACAGCATGTCCTAGCAAGCCAGAATTCGGCAGCGT<br>UAGCUAUCAGAGAUCUGUCGUACAGG 5'
3'RNA$_{50}$:RNA$_{26}$	5' AUCGAUAGUCUCUAGACAGCAUGUCCUAGCAAGCCAGAAUUCGGCAGCGU<br>UAGCUAUCAGAGAUCUGUCGUACAGG 5'
5'RNA$_{50}$:RNA$_{26}$	5' AUCGAUAGUCUCUAGACAGCAUGUCCUAGCAAGCCAGAAUUCGGCAGCGU<br>GGAUCGUUCGGUCUUAAGCCGUCGCA 5'
