# Supplementary figures and images for: Phylogenetic Diversity of Lhr Proteins and Biochemical Activities of the Thermococcales aLhr2 DNA/RNA Helicase
Source: Biomolecules. 2021 Jun 26;11(7):950. doi: 10.3390/biom11070950 (PMC8301817; doi:10.3390/biom11070950)

Figure S1

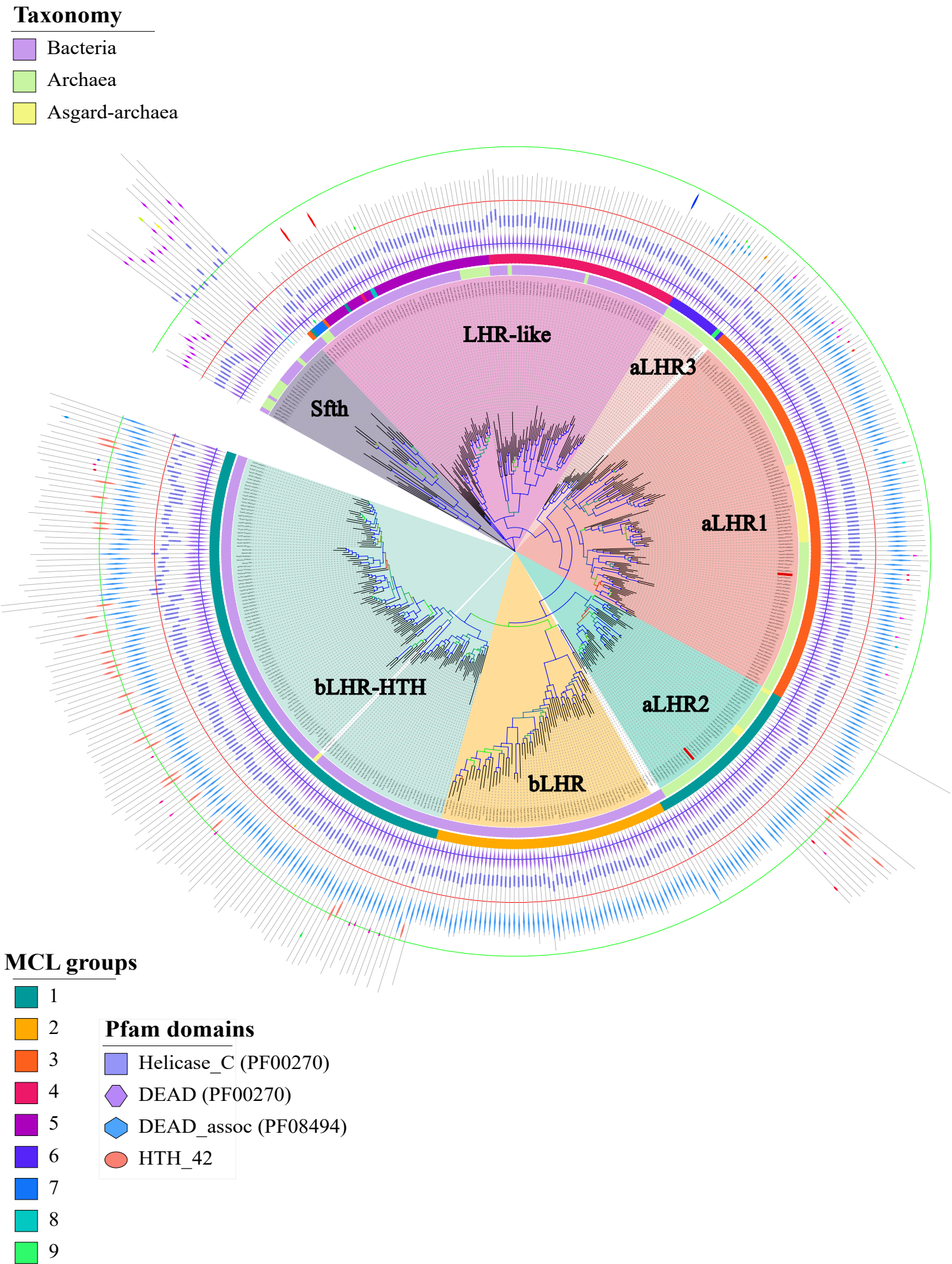

Supplement: Supplementary file 1 [file biomolecules-11-00950-s001.zip › FIGURE S1 v18-06.pdf]

Figure S2

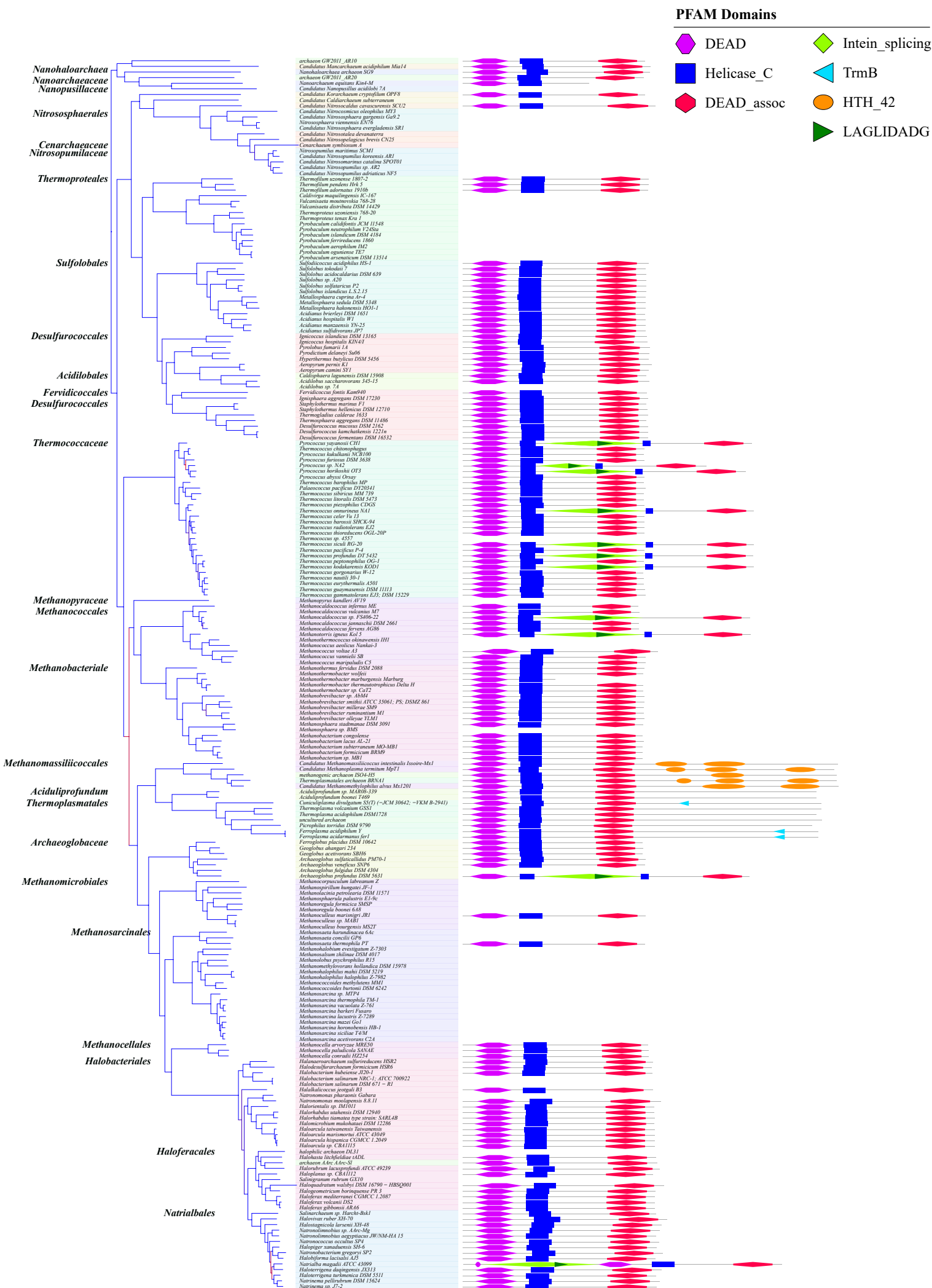

Supplement: Supplementary file 1 [file biomolecules-11-00950-s001.zip › FIGURE S2 v18-06.pdf]

Figure S3

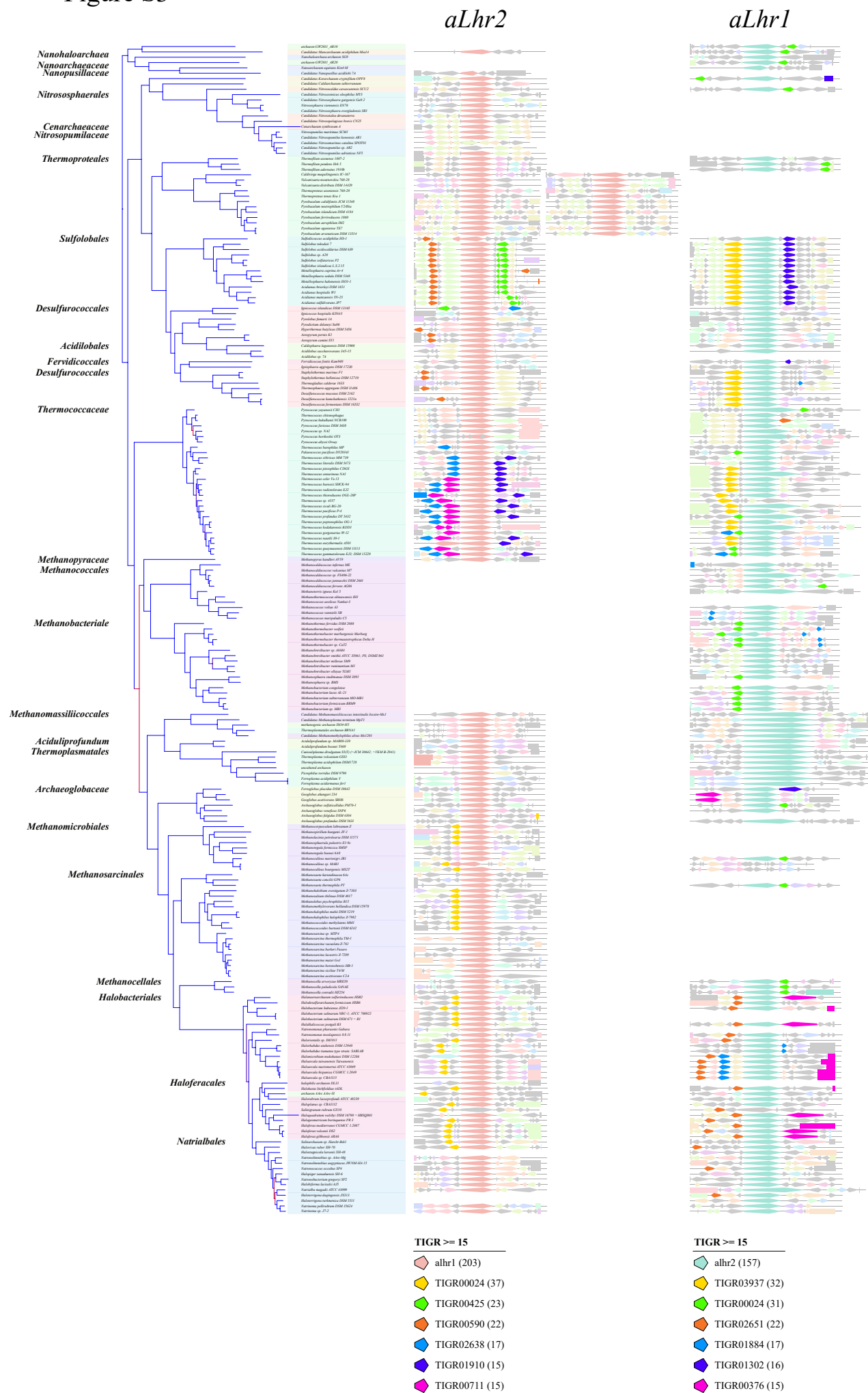

Supplement: Supplementary file 1 [file biomolecules-11-00950-s001.zip › FIGURE S3 v18-06.pdf]

Figure S4

A.

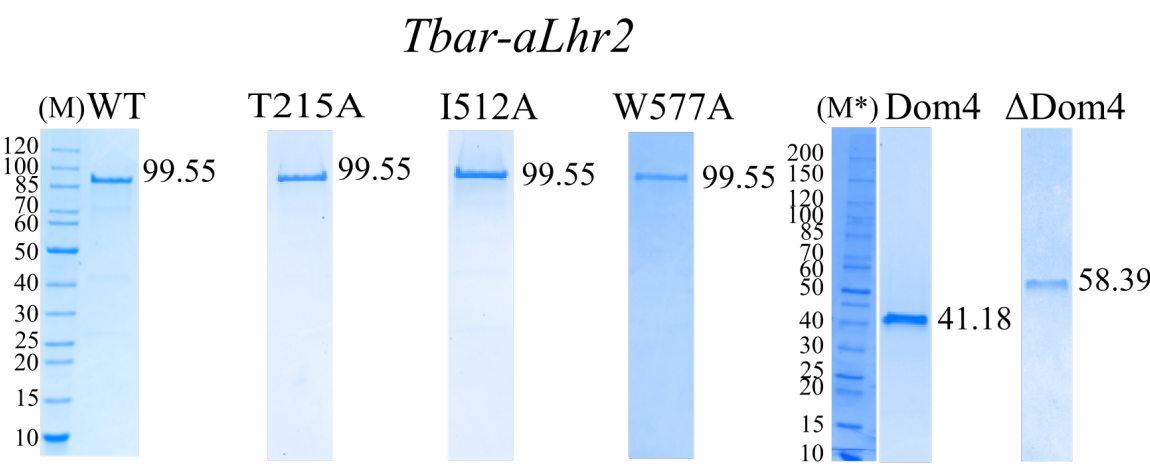

B.

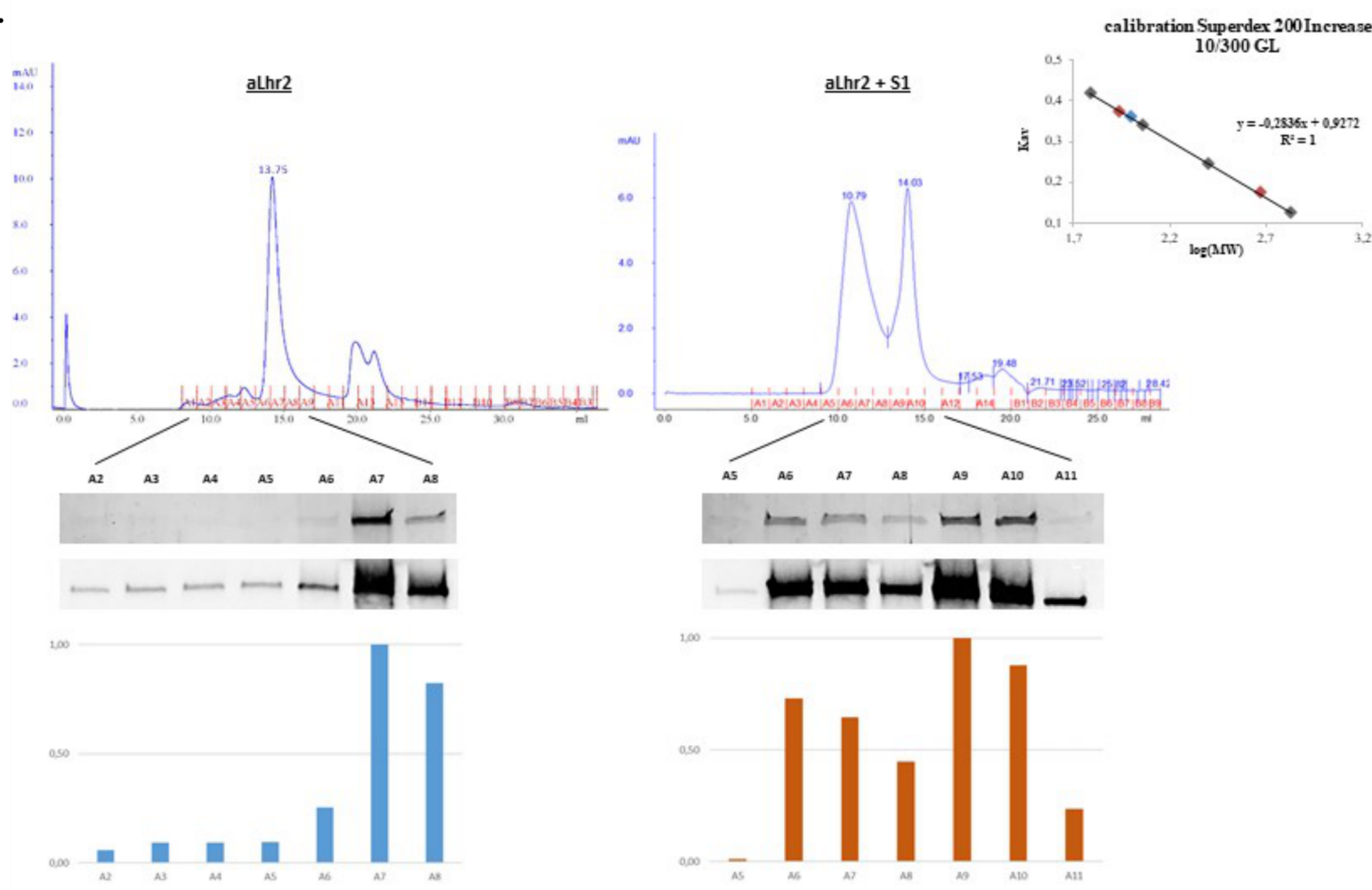

Supplement: Supplementary file 1 [file biomolecules-11-00950-s001.zip › FIGURE S4 v18-06.pdf]

Figure S5

A.

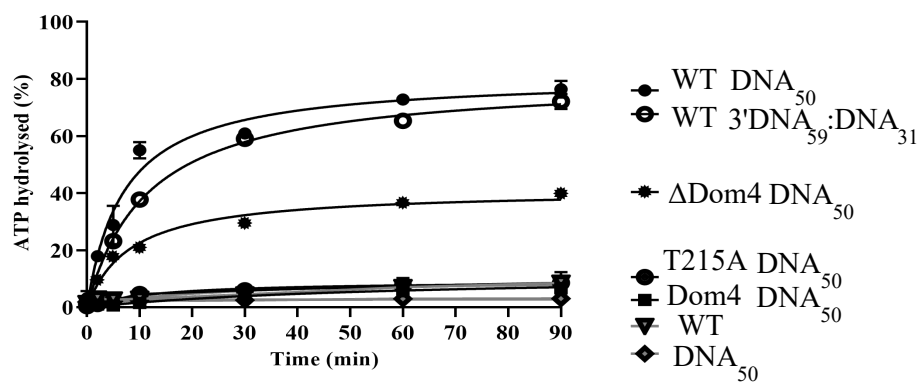

B.

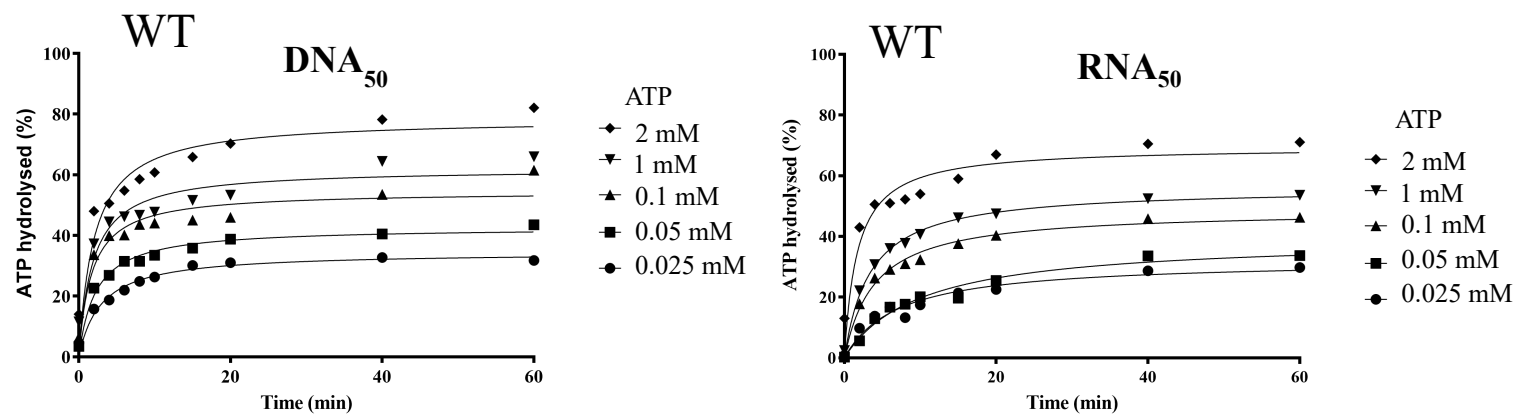

C.

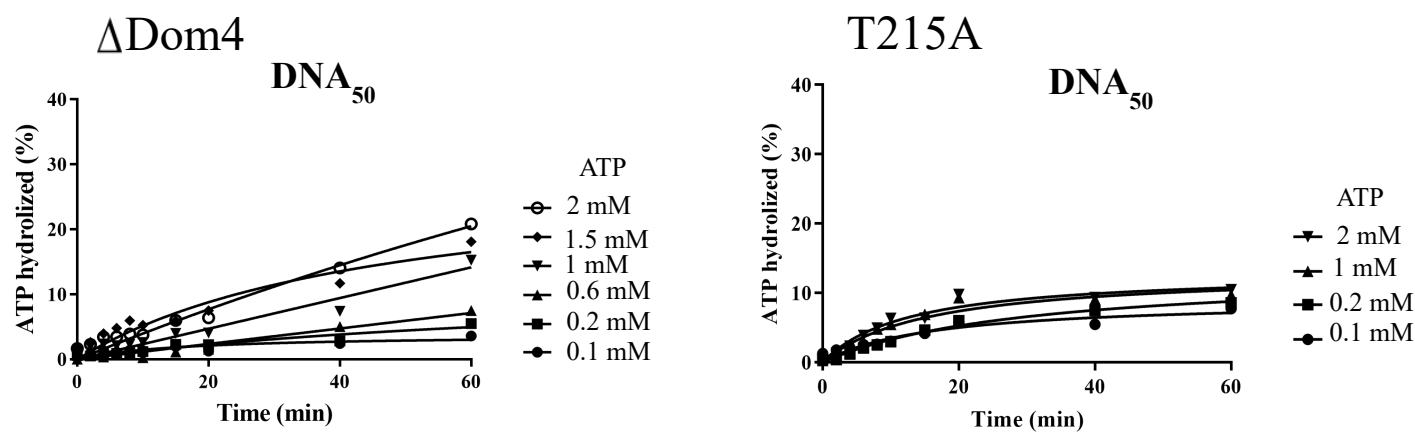

Supplement: Supplementary file 1 [file biomolecules-11-00950-s001.zip › FIGURE S5 v18-06.pdf]

Figure S6

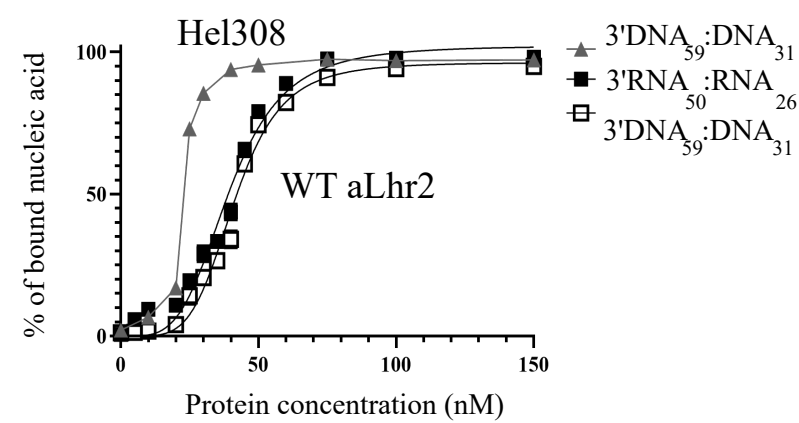

Supplement: Supplementary file 1 [file biomolecules-11-00950-s001.zip › FIGURE S6 v18-06.pdf]

Figure S7

A.

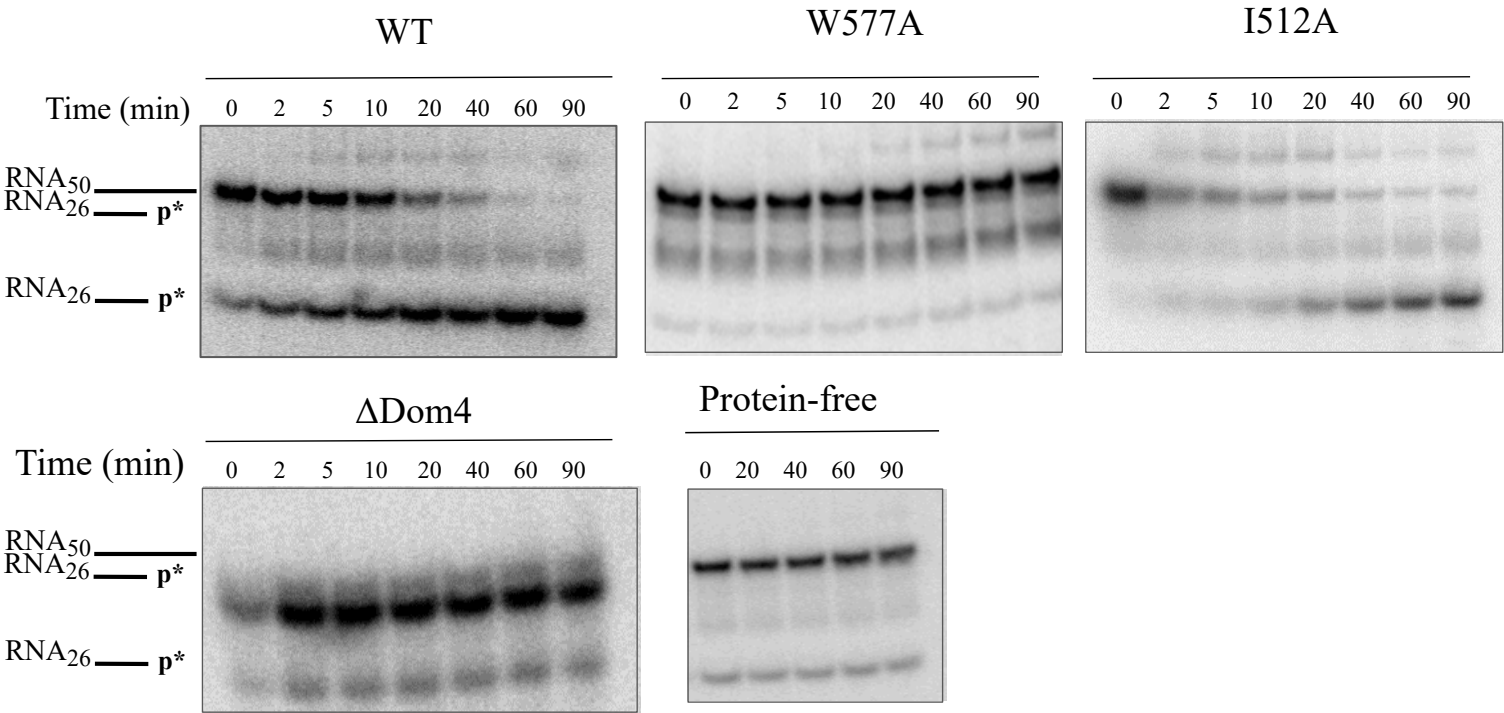

B.

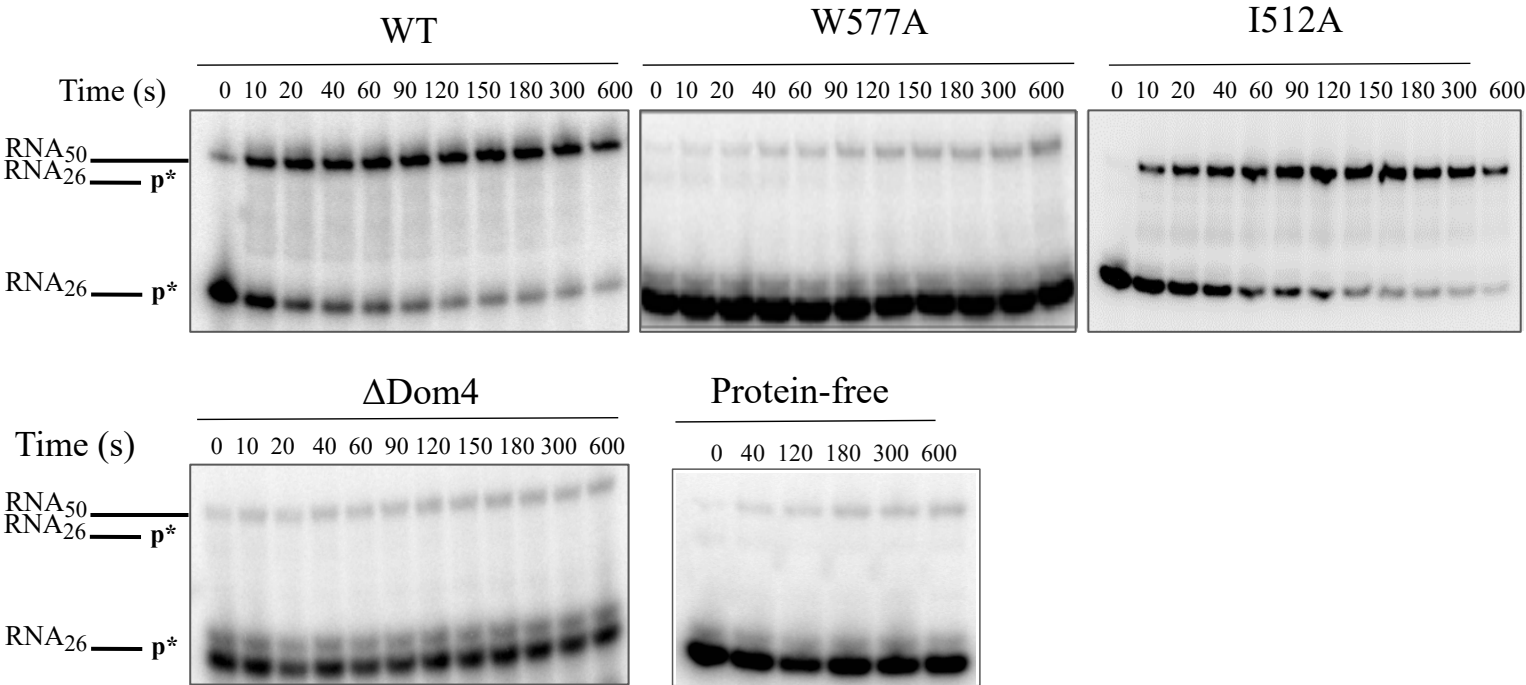

Supplement: Supplementary file 1 [file biomolecules-11-00950-s001.zip › FIGURE S7 V18-06.pdf]

Figure S8

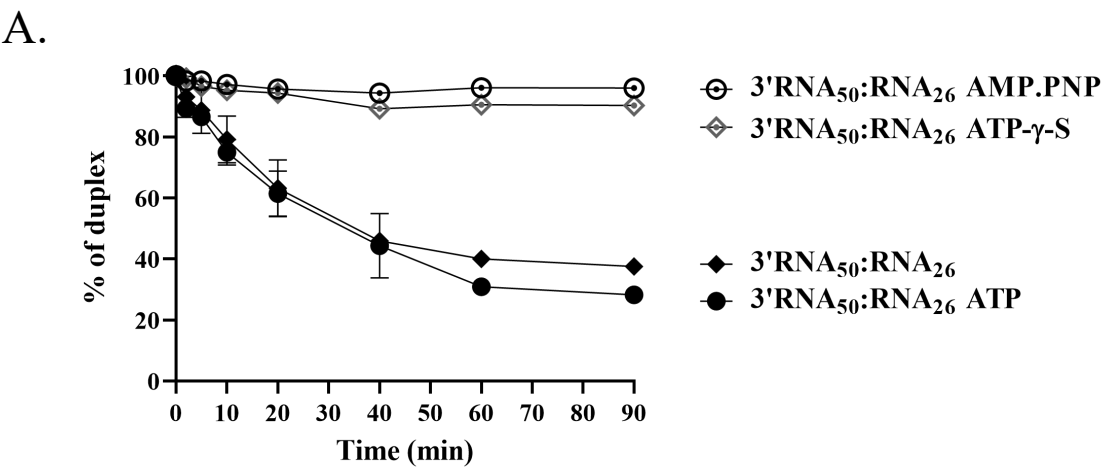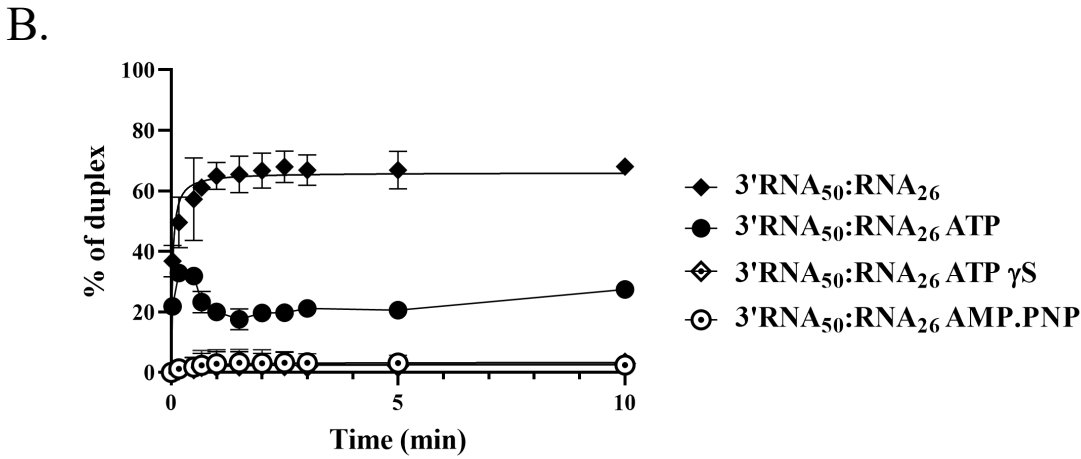

Supplement: Supplementary file 1 [file biomolecules-11-00950-s001.zip › FIGURE S8 v18-06.pdf]

Figure S9

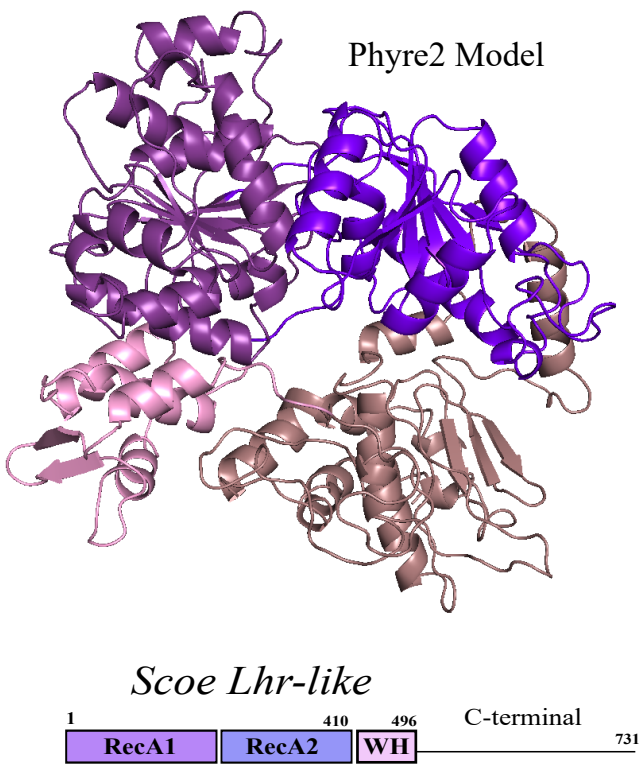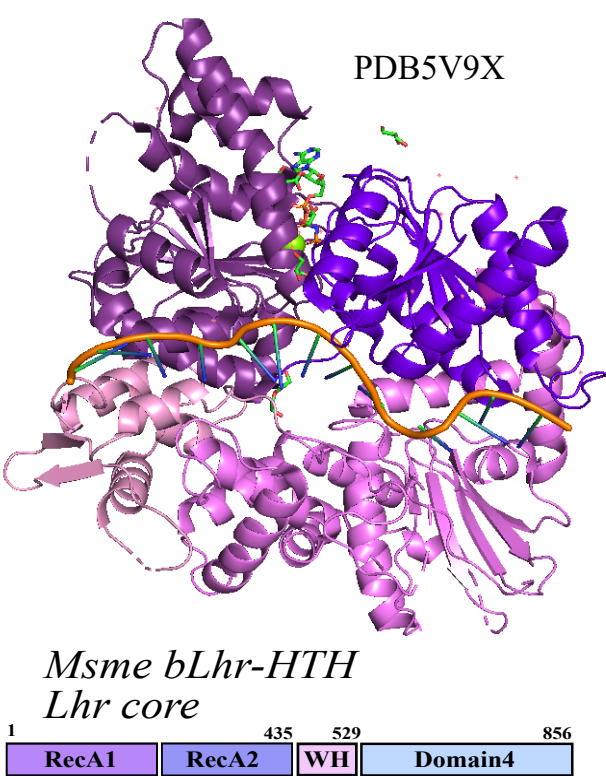

Supplement: Supplementary file 1 [file biomolecules-11-00950-s001.zip › FIGURE S9 v18-06.pdf]
